# TRPM8 Puts the Chill on Prostate Cancer

**DOI:** 10.3390/ph9030044

**Published:** 2016-07-09

**Authors:** Guillaume P. Grolez, Dimitra Gkika

**Affiliations:** Laboratoire de Physiologie cellulaire, Inserm U1003, Laboratory of Excellence, Ion Channels Science and Therapeutics, Université de Lille, 59655 Villeneuve d’Ascq Cedex, France; guillaume.grolez@etudiant.univ-lille1.fr

**Keywords:** TRPM8, prostate cancer, migration

## Abstract

Prostate cancer (PCa) is one of the most frequently diagnosed cancers in developed countries. Several studies suggest that variations in calcium homeostasis are involved in carcinogenesis. Interestingly, (Transient Receptor Potential Melastatin member 8) TRPM8 calcium permeable channel expression is differentially regulated during prostate carcinogenesis, thereby suggesting a potential functional role for this channel in those cell processes, which are important for PCa evolution. Indeed, several studies have shown that TRPM8 plays a key role in processes such as the proliferation, viability and cell migration of PCa cells. Where cell migration is concerned, TRPM8 seems to have a protective anti-invasive effect and could be a particularly promising therapeutic target. The goal of this review is to inventory advances in understanding of the role of TRPM8 in the installation and progression of PCa.

## 1. Introduction

In developed countries, prostate cancer is the second most frequently diagnosed cancer and the third most common cause of death by cancer in men [1]. Prostate cancer (PCa) development starts from epithelial cells in the peripheral zone of the prostate and is androgen-controlled [2]. In its first stages, the cancer develops slowly and remains localized, while in later stages the prostate capsule barrier can be crossed, and PCa becomes invasive, often leading to metastasis in lymph nodes and later mainly in the bone, liver and lung [3]. Metastasis development in the late PCa stages is the main cause of mortality due to PCa. To prevent PCa development, the main treatment is tumor ablation followed by hormone-therapy and more precisely androgen suppression. This treatment is the leading treatment in the case of metastasis, and currently the most successful since it leads to tumor regression. However, PCa cells can become androgen-independent for their survival and proliferation and therefore escape this treatment, leading to more aggressive forms of cancer [4]. This androgen insensitivity is a significantly increases PCa mortality rates and suggests that various androgen-related factors are involved in PCa progression.

Several studies suggest that variations or modifications to Ca^2+^ homeostasis are involved in PCa carcinogenesis and in metastasis development of since this affects the key cellular processes of carcinogenesis [5,6,7,8]. Indeed, malignant cell-transformation is the result of enhanced proliferation, aberrant differentiation, and an impaired ability to die [9]. These result in abnormal tissue growth, which can eventually turn into the uncontrolled expansion and invasion that is characteristic of cancer. Such transformation is often accompanied by changes in ion channel expression and, consequently, by the abnormal progression of the cellular responses with which they are involved. Members of TRP (Transient Receptor Potential) ion channel superfamily are implicated in all the hallmarks of cancer, while their expression levels are correlated with the emergence and/or progression of numerous epithelial cancers [10,11,12,13,14]. In addition, it should be noted that besides their transcriptional and translational regulation, ion channel trafficking to the cell surface as well as plasma membrane stabilization define channel activity. Therefore, modulation of TRP expression/activity on one of these levels affects intracellular Ca^2+^ concentrations and, consequently, those processes involved in carcinogenesis, such as proliferation, apoptosis, and migration [12].

## 2. TRPM8: An Androgen Target in Prostate Cancer

TRPM8 is one of the TRP channels involved in PCa and it seems to be one of the most promising clinical targets. Its androgen-dependent expression increases in both benign prostate hyperplasia and in prostate carcinoma cells, which both presented high androgen levels [15], while anti-androgen therapy greatly reduces the expression of TRPM8 [16]. It appears that the androgen dependency of TRPM8 expression is related to the differentiation degree of prostate epithelial cells [17,18]. Recent studies suggest that androgens could act in a non-genomic way on TRPM8 channel [19].

Furthermore, androgens have been shown to define the subcellular localization of TRPM8 toward different healthy and cancerous prostate cells [18]. Several studies have demonstrated both plasma membrane and endoplasmic reticulum (ER) membrane TRPM8 expression, in prostate cancer cell lines that are androgen-sensitive, such as LNCaP cells (Lymph Node Carcinoma of the Prostate) [20,21]. TRPM8 expression at the plasma membrane seems to increase in correlation with the increase of functional AR expression, while the ER isoform is less sensitive to androgens. At the functional level, the ER isoform has been shown to be involved in the activation of store-operated channels [20]. Thus, dual localization of TRPM8 in the two membranes significantly increases the spectrum of physiological and pathological processes the channel may be involved in. Indeed, plasma membrane or ER ion channels localization induce different calcium signaling patterns, which are responsible for the inception of various cellular processes. For example, when TRPM8 channel localizes at the plasma membrane, it is mainly involved in cancer proliferation and migration by activation of various calcium dependent pathways [22,23], whereas ER ion channel localization has been shown to be involved in the balance between apoptosis and proliferation [8,24]. These processes have been defined as being hallmarks of cancer and TRPM8 plays a role in all of these. Among these hallmarks, cell migration is the most involved in the evolution of cancer towards metastatic stages.

## 3. Roles of TRPM8 in PCa Progression

Malignant transformation of cells is the result of enhanced proliferation, aberrant differentiation, and an impaired ability to die, which results in tumor growth and potential invasion of the surrounding tissues and eventually metastasis [9]. This transformation is characterized by changes in the ion channel expression profile, which in turn modifies the cellular responses they are involved in. Different studies have shown an involvement of the TRPM8 channel in these processes in PCa cells and more particularly, TRPM8 has been shown to play a major role in migration.

### 3.1. Role of TRPM8 in Proliferation

The role of TRPM8 in the proliferation of PCa cells was shown using in vitro assays measuring cell viability, in cell cycle assays and with in vivo studies. Firstly, an anti-proliferative effect of TRPM8 on PCa cell lines was demonstrated using in vitro assays. Indeed, in androgen insensitive PCa cells (DU-145), endogenous TRPM8 activation by menthol treatment induces a decrease in proliferation [25]. Moreover, over-expression of TRPM8 in PC3 androgen insensitive cells that do not express TRPM8 at endogenous level induces a decrease in proliferation. To mediate this anti-proliferation effect, TRPM8 over-expression induces an arrest in the cell cycle from the G1 to S phase transition. This arrest is due to a down-regulation of Cdk4 and Cdk6 proteins after TRPM8 overexpression [26]. An in vivo study also showed an anti-proliferative effect of TRPM8 over-expression in PCa cells. Indeed, tumors monitored by xenograft in mice with PC3 cells overexpressing TRPM8, were less voluminous than tumors in PC3 cells, which were not [27].

On the other hand, a pro-proliferative role was shown in vitro for TRPM8 by using blockers and siRNA against TRPM8 [28]. In this study, the authors tested the effects of TRPM8 inhibition on various PCa cell lines. TRPM8 blockers and siRNA were shown to reduce the proliferation of LNCap cells and DU-145 cells, but not in PC3 cells and PNTA1 cells. The different effects of TRPM8 siRNA on PCa cell proliferation could not be explained by the difference in TRPM8 expression because TRPM8 was be shown to expressed at the same level in both PC3 and LNCaP cells [29].

The discrepancy in the TRPM8 effect on proliferation mentioned above could be explained by the distinct androgen sensitivity of the cell lines used. In fact, TRPM8 seems to play an anti-proliferative role in PCa androgen insensitive cells (PC3 and DU-145), yet a pro-proliferative role in PCa androgen sensitive cells (LNCaP). This result suggests that TRPM8 channels could be useful PCa proliferation arrest targets in the first stage of cancer, when pharmacological blockers could be used.

### 3.2. Role of TRPM8 in Cell Death or Survival

During cancer progression, cell survival balance is disturbed, thereby causing a resistance to cell death and a resistance to apoptosis in particular. Various studies have shown an interest in the TRPM8 role in cell viability in the PCa. Indeed, by its ER localization, TRPM8 was shown to be involved in the activation of store-operated channels [20], inducing an increase in cytosolic calcium concentration. Moreover, in LNCaP cell lines, ER store depletion has been shown to be sufficient to induce the apoptosis process [30] suggesting a possible role for TRPM8 in the apoptosis balance. Different studies seem to show a pro-apoptotic role of TRPM8 in PCa cells. In vitro assays using flow cytometry on PC3 cells show that TRPM8 overexpression increases apoptosis rates in starved conditions (1% FBS for 48 h). Moreover, as previously mentioned, TRPM8 overexpression in these cells induces arrest of the cell cycle in the G0/G1 stages [26] TRPM8 has also been shown to have a pro-apoptotic role in DU-145 cells after activation by menthol [25]. Finally, sustained activation of TRPM8 by menthol induces an increase in apoptosis in LNCaP cells due to the increase in the cytosolic calcium concentration [21].

On the other hand, one study using TRPM8 siRNA and blockers showed the opposite effects. Indeed, in LNCaP cells, siRNA-mediated knockdown of TRPM8, or a capsazepine treatment induced an apoptotic process shown by the increased numbers of cells with apoptotic nuclei [21]. By this study, authors show that TRPM8 are necessary for the survival and the anti-apoptotic role of TRPM8 in LNCaP cells. Another interesting study shows an anti-apoptotic role of a short isoform of TRPM8 expressed in PCa cells [31]. Overexpression of the short TRPM8 isoform, sM8a, reduces the apoptosis induced by starvation in LNCaP cells. This sM8a isoform is a 19 kDa protein, which was previously shown to negatively regulate the full length of TRPM8 by interaction [32].

Overall, these studies show that TRPM8 plays a role in cell viability, which seems to be regulated by androgens as well as the differential expression of TRPM8 isoforms expressed in PCa cells.

### 3.3. Role of TRPM8 in Migration

As mentioned above, metastasis development is the main cause of cancer-related mortality, and depends on two key processes: cell migration of cancer cells that invade adjacent tissues, followed by intravasation into blood/lymphatic vessels and tumor vascularization, which give access to the bloodstream. During the metastatic process, cell migration of both epithelial and endothelial cells is an essential step leading to the spread of the primary tumor and to the invasion of neighboring connective tissue, the lymphatic system and blood vessels. The role of TRPM8 channel in the PCa migration process in has been studied recently. Indeed, we as well as others suggest a putative protective role for TRPM8 in prostate metastatic cancer progression [33], since enhancement in channel expression and/or an activation, blocks prostate cancer cell migration [22,26,27]. In this context, we have shown that PCa cell treated with icilin, a TRPM8 agonist, results in a decrease in the cell mobility of PC3 cells overexpressing TRPM8 [22]. In line with these results, two other studies demonstrate that TRPM8 overexpression significantly inhibits PC3 cell migration and they show that this inhibition occurred through the inactivation of focal adhesion kinase (FAK) [26,27]. FAK is the non-receptor protein, tyrosine kinase, the phosphorylation of which is critical during focal adhesion formation and consequently in cellular processes such as migration and invasion [34]. These studies showed that TRPM8 activation by different agonists or overexpression of TRPM8 channels induces an inhibition of PCa migration. Nevertheless, it has to be noted that contrasting results concerning the role of TRPM8 in cell migration have been shown using pharmacological agents inhibiting TRPM8, which have led to a reduction in the speed of prostate cancer cells [28,35].

Further, TRPM8-mediated cancer cell migration has been seen to be regulated by the newly identified partner proteins of the channel. Firstly, Prostate Specific Antigen (PSA), shown to be an endogenous agonist of TRPM8 increasing the channel activity while supporting its plasma membrane expression, was shown to induce a decrease in cell mobility in PC3 cells overexpressing TRPM8 [22]. Moreover, the channel-associated protein TCAF1, which is also strongly expressed in the prostate [36], was shown to facilitate the opening state of TRPM8 and plasma membrane expression by direct interaction. This protein, by activating TRPM8 regulation, was shown to decrease the migration of PCa cells by reducing both cell speed and velocity [36]. In line with these results, the short TRPM8 isoform sM8a, acts as a partner protein to the channel inhibiting full length TRPM8 and promoting cell mobility and invasion when overexpressed in LNCaP cells [31].

In summary, taking into account the aforementioned studies using endogenous and exogenous agonists of the TRPM8 channel, as well as the overexpression of this protein in PCa cells, one can conclude that there is an anti-migratory role of this channel in PCa cells. Since migration is one of the key processes of metastatic development, these results suggest a protective role for TRPM8 in prostate metastatic cancer progression. To further confirm this hypothesis, Zhu et al. were interested in the role of TRPM8 on angiogenesis, which is also a key process for metastasis development. An in vivo study in nude mice showed that TRPM8 expression had a negative effect on angiogenesis [27]. Indeed, mice transplanted with prostate cancer cells over-expressing TRPM8 develop tumors that are less vascularized than control cases. The lower micro-vascular density of the TRPM8 xenografts can be explained by their lower expression of FAK and VEGF, which is one of the most potent angiogenic factors [27]. Taken together, these results reinforce the hypothesis that TRPM8 could play a protective role in prostate cancer progression by reducing both cell migration and angiogenesis.

## 4. Discussion

Several lines of evidence have been discovered over recent decades showing the importance of TRPM8 in prostate cancer. Firstly, it has been demonstrated that expression of TRPM8 varies during cancer progression and the androgen-dependence of TRPM8 expression has been demonstrated. A loss of TRPM8 expression is positively correlated with the aggressive androgen-independent state of PCa. Indeed, TRPM8 is strongly expressed in the first PCa stages and its expression disappears in the late and more aggressive states of the PCa. By this variation in its expression, TRPM8 may be considered and used as a diagnostic/prognostic marker as it is already used as a clinical marker in some countries [37,38].

Several studies cited in this review have shown by the use of agonists and blockers of TRPM8 that this channel has mainly anti-proliferative, pro-apoptotic and anti-migratory roles in PCa cells. As proliferation and cell viability data are contradictory, further experiments are needed and particularly in vivo data in order to confirm an anti-cancer role for this channel. However, there is less controversy concerning the TRPM8 anti-migratory role, consequently suggesting a protective role for this channel against metastasis in PCa. This is why several regulatory agents (listed in Table 1) could be used to prevent the metastatic evolution of PCa when the cancer is diagnosed.

Another way to prevent the metastatic evolution of PCa is to reinforce the activation of TRPM8 endogenous regulator proteins (listed on Table 2) and to target the channel’s activity. These different endogenous or pharmacological modulators of TRPM8 are very promising for the prevention of the metastatic evolution of PCa and should therefore be validated in animal models.

## 5. Conclusions

In conclusion, TRPM8 variation in expression during the evolution of PCa makes this channel an appealing clinical marker in order to separate the different stages of the disease. Concerning the therapeutic targeting of the channel, at first sight the evolution of TRPM8 expression during carcinogenesis might seem not in full accordance with the anti-cancer effect of the channel activation. In order to make sense of TRPM8’s role in prostate carcinogenesis, the different hallmarks of cancer should be studied separately; at the beginning, by taking into account the androgen dependency of the PCa cells in the various stages of advancement. However, the results till now support the hypothesis that TRPM8 increase in the first androgen-dependent stages of PCa has a protective role regarding the invasive character of prostate cancer cells. Indeed, to support this hypothesis, a preclinical assay with a TRPM8 agonist (D-3263) show that TRPM8 activation decrease mice prostate hyperplasia [45]. Finally, for these early stages, how the high expression of the channel is functionally related to the growth of the tumor volume remains to be elucidated.

## Figures and Tables

**Table 1:** TRPM8 modulators can be used in research therapies against PCa.

Molecule	Agonist/Antagonist	PCa-Related Cellular Effect
Capsazepine [21]	Antagonist	Increased apoptosis in LnCaP cells
AMTB [28,39]	Antagonist	Decreased proliferation in LnCaP cells
JNJ-39267631 [40]	Antagonist	Not defined
BCTC [28]	Antagonist	Decreased cell proliferation
M8-B [41]	Antagonist	Not defined
Cannabigerol [42]	Antagonist	Pro-apoptotic effects in Pca cells
PBMC [43]	Antagonist	Not defined
PSA [22]	Agonist	Decreased cell mobility in PC3-TRPM8 cells
Icilin [22]	Agonist	Decreased cell mobility in PC3-TRPM8 cells
Menthol [21,25]	Agonist	Decreased proliferaion, increased apoptosis
WS12 [44]	Agonist	Not defined
D-3263 [45]	Agonist	Decrease mice prostate hyperplasia

**Table 2:** TRPM8 regulatory proteins can be used in research therapies against PCa.

Partners Protein	Agonist/Antagonist	PCa-Related Cellular Effect
G alpha protein [46]	Antagonist	Inhibition of TRPM8
sM8a protein [32]	Antagonist	Negative regulation of full length TRPM8
Pirt [47]	Agonist	Enhances TRPM8 channel properties
TCAF1 [36]	Agonist	Facilitates the opening state of TRPM8 and plasma membrane expression
PYR-41 [48]	Agonist	Facilitates TRPM8 plasma membrane expression
